# Exploring Darunavir, Rilpivirine and Etravirine as Potential Therapies for Bladder Cancer: Efficacy and Synergistic Effects

**DOI:** 10.3390/biomedicines12030647

**Published:** 2024-03-14

**Authors:** Mariana Pereira, Nuno Vale

**Affiliations:** 1 PerMed Research Group, Center for Health Technology and Services Research (CINTESIS), Rua Dr. Plácido da Costa, 4200-450 Porto, Portugal; mariana.m.pereira2097@gmail.com; 2 CINTESIS@RISE, Faculty of Medicine, University of Porto, Alameda Professor Hernâni Monteiro, 4200-319 Porto, Portugal; 3 ICBAS—School of Medicine and Biomedical Sciences, University of Porto, Rua de Jorge Viterbo Ferreira, 228, 4050-313 Porto, Portugal; 4 Department of Community Medicine, Information and Health Decision Sciences (MEDCIDS), Faculty of Medicine, University of Porto, Alameda Professor Hernâni Monteiro, 4200-319 Porto, Portugal

**Keywords:** bladder cancer, antiretroviral drugs, efficacy evaluation, etravirine, synergy, repurposing possibilities, personalized medicine

## Abstract

This research explores the therapeutic efficacy of Darunavir (DRV), Rilpivirine (RPV), and Etravirine (ETV) against UM-UC-5 bladder cancer cells, addressing the critical need for innovative treatments in bladder cancer research. Through a comprehensive assessment of their individual and combined effects across diverse time intervals, ETV emerges as the most potent drug, with a lowest IC_50_ of 5.9 µM, closely followed by RPV (lowest IC_50_ of 9.6 µM), while DRV exhibits the least effectiveness (lowest IC_50_ of 25.6 µM). Notably, a significant synergistic effect is evident in the ETV and RPV combination, especially at 48 and 72 h for low concentrations. Synergies are also observed with ETV and DRV, albeit to a lesser extent and primarily at 48 h. Conversely, the DRV and RPV combination yields minimal effects, predominantly additive in nature. In summary, this pre-clinical investigation underscores the promising therapeutic potential of ETV and RPV, both as standalone treatments and in combination, hinting at repurposing opportunities in bladder cancer therapy, which could give a new treatment method for this disease that is faster and without as severe side effects as anticancer drugs. These findings represent a substantial stride in advancing personalized medicine within cancer research and will be further scrutinized in forthcoming studies.

## 1. Introduction

Bladder cancer (BC) is the most common urinary tract cancer type, with more than half a million new cases and 200,000 deaths worldwide in 2020 [1]. It has a higher incidence and mortality in Asia and Europe, with males being generally more affected than females [1,2]. Bladder cancer is associated with 90–95% of cancerous alterations in urothelial “umbrella” cells (urothelial carcinoma), which, in this case, line the urinary bladder lumen (although urothelial cancers also encompass carcinomas in other parts of the urinary tract). Of all cases, 75% are histologically pure carcinomas and the others have altered histologic profiles [3]. Bladder cancer has been associated with cardiovascular disease (CVD), as a study shows a positive relationship between the incidence of bladder cancer and cardiovascular disease (CVD), with CVD acting as an independent protective factor against bladder cancer without affecting tumor aggressiveness. The varying effects of comorbidities on individuals with bladder cancer, especially concerning tumor staging, point to a complicated interaction between risk factors, tumor histotypes, and genetics [4].

Among the aforementioned histologic variants of urothelial carcinomas is the squamous cell carcinoma of the bladder (SCC-BC), which accounts for around 2–5% of carcinomas. SCC-BC can be associated directly with having a bilharziasis infection (B-SCC-BC) or not (NB-SCC-BC) [5]. This disease, also named schistosomiasis, is a parasitic infection with trematode worms and occurs mainly in the developing countries of Africa, the Middle East, and South America [6]. B-SCC-BC will be detected earlier in life and is associated mainly with the urinary bacterial infections derived from bilharziasis and not with the parasite itself. These will have carcinogenic outcomes by producing the enzyme β-glucuronidase that metabolizes conjugated carcinogens into free carcinogens, as well as by the direct production of carcinogenic nitrosamines [7].

Non-bilharziasis SCC-BC (NB-SCC-BC) is more common in Western countries and is connected to being an older age. It is often associated with people who have chronic bladder inflammatory diseases, chronic cystitis, and persistent calculi, with a marked association between patients with spinal cord injuries and NB-SCC-BC due to chronic urinary tract inflammation. NB-SCC-BC will be muscle invasive when diagnosed, having a poor diagnosis of 1–3 years of survival, with the better treatment being a radical cystectomy. Typically, this carcinoma will present as a large tumor that will involve the bladder wall and neighboring areas of the squamous metaplasia and ulceration [8].

This is a disease that still needs more treatment methodologies, as bladder cancer has several molecular subtypes with different pathways of disease, some with high mutational genes [9]. Bladder cancer treatment is mostly based on high-cost, invasive procedures, and targeted therapies to specific upregulated pathways are urgent [10]. This drug repurposing, which involves administering medications that have already obtained approval but for a different application, is an emerging strategy that may be useful.

The combination of repurposed drugs either with other repurposed drugs or with antineoplastic drugs can heighten the effect of drugs even more, and allows for a decrease of individual doses, with an accompanied decrease of secondary effects, and even for overcoming drug resistance [11]. In this article, three antiretroviral drugs are studied to understand if these can be repurposed for squamous bladder cancer. Human squamous cell carcinoma of the bladder, UM-UC-5, is a human transitional cell carcinoma of the bladder. These cells were studied for their susceptibility to tumor growth in nude mice and differences in genetic alterations. In a pharmacological evaluation, these assays allowed for the characterization of some of the most important features of carcinoma of the bladder. Furthermore, they may be equally useful to more accurately establish commonly observed phenomena across cells of the same type of neoplasm. Antiretrovirals were chosen as the focus of this study since it has been shown that there is a correlation between antiretroviral treatment and positive outcomes of cancer treatment in low- and middle-income countries [12]. In the current study, the aim was to test three antiretrovirals, Darunavir, Rilpivirine, and Etravirine, and a quick overview of these drugs is given below.

Darunavir (DRV) is a nonpeptidyl small molecule that acts as an HIV protease inhibitor (PI) and is typically used for the treatment of HIV in combination with other drugs, particularly for multi-experienced patients. This drug inhibits the dimerization and activity of the HIV protease, which is then incapable of cleaving the *gal-pol* polyproteins needed for virion maturation [13]. This drug was developed by Janssen Pharmaceuticals and was commercialized under the name Prezista^®^ (Janssen Pharmaceuticals, Beerse, Belgium) in 2006, with generics already existing [14].

Rilpivirine (RPV) is a second-generation non-nucleoside reverse transcriptase inhibitor (NNRTI) antiretroviral medication with a diarylpyrimidine derivative that is used to treat HIV. Following the US Food and Drug Administration’s (FDA’s) and the European Medicines Agency’s (EMA’s) clearance in 2011 [15,16], Janssen Pharmaceuticals (Beerse, Belgium) has been producing this medication under the brand name Edurant^®^ (Janssen Pharmaceuticals, Beerse, Belgium). RPV works by directly interacting with the HIV reverse transcriptase (RT) allosteric site through RPV’s cyanovinyl group, altering the shape of the nucleic acid binding cleft. As a result, the nucleosides are unable to attach to the reverse transcriptase, which prevents the cDNA elongation process from continuing, something crucial for HIV infection [17].

**Figure 1:**
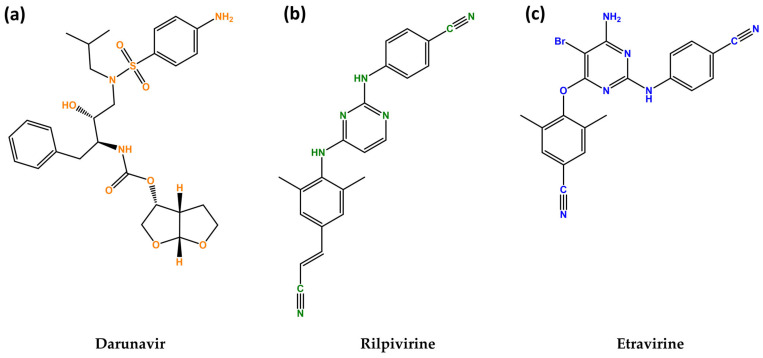
Chemical structures of darunavir (DVR) (**a**), rilpivirine (RPV) (**b**) and etravirine (ETV) (**c**). Developed with ChemBioDraw^®^ Ultra version 13.0. A Chemical Drawing Software. Available online: https://chemdrawdirect.perkinelmer.cloud/js/sample/index.html (accessed on 10 July 2023).

The last drug of this study is etravirine (ETV), which is also a second-generation diarylpyrimidine NNRTI that was approved in 2007 in the USA under the name Intelence^®^ (Janssen Pharmaceuticals, Beerse, Belgium) and is used in combination for the treatment of treatment-experienced patients [18]. ETV inhibits both RNA- and DNA-dependent polymerase activities allosterically by binding in a pocket next to the catalytic site of reverse transcriptase. This prevents the synthesis of viral cDNA (copy DNA). Additionally, etravirine impacts post-integration stages, possibly by improving the processing of the precursor proteins *gag* and *gag-pol* in HIV-1 transfected cells, which reduces the production of viral particles [19]. The chemical structures of these drugs are illustrated in Figure 1.

The present study aimed to study these three drugs, DRV, RPV, and ETV, alone and in combination with each other in squamous bladder cancer cells to evaluate their cytotoxicity as well as their combination relationship.

## 2. Results

### 2.1. Drugs Alone

#### 2.1.1. Cytotoxicity of Darunavir

**Figure 2:**
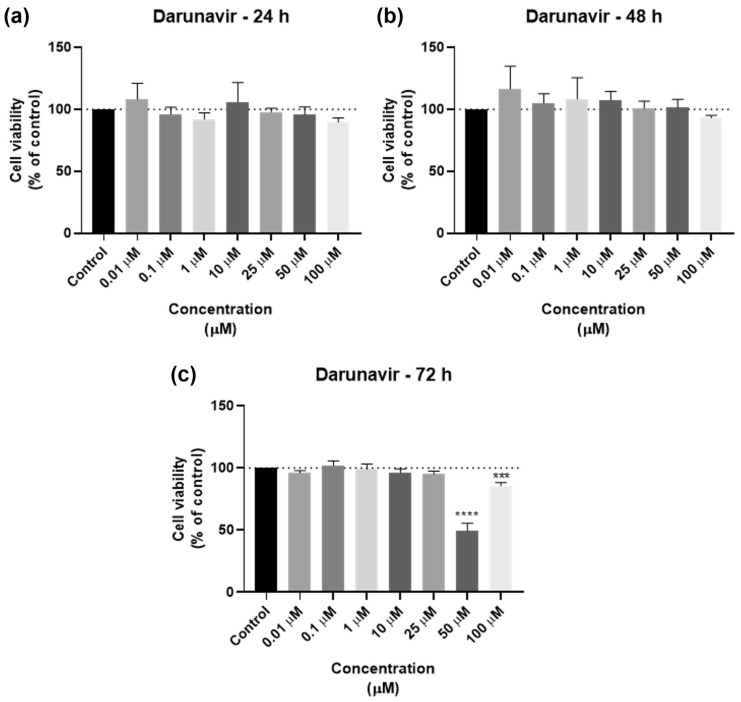
Results of UM-UC-5 cell viability following exposure to DRV at escalating doses (0.01–100 µM) for 24 h (**a**), 48 h (**b**), and 72 h (**c**). A 0.1% DMSO was applied to negative control cells (vehicle). The MTT assay was used to determine cell viability, and the findings are shown as the mean ± SEM (*n* = 3). *** Statistically significant vs. negative control (vehicle) at *p* < 0.001; **** Statistically significant vs. negative control (vehicle) at *p* < 0.0001.

**Figure 3:**
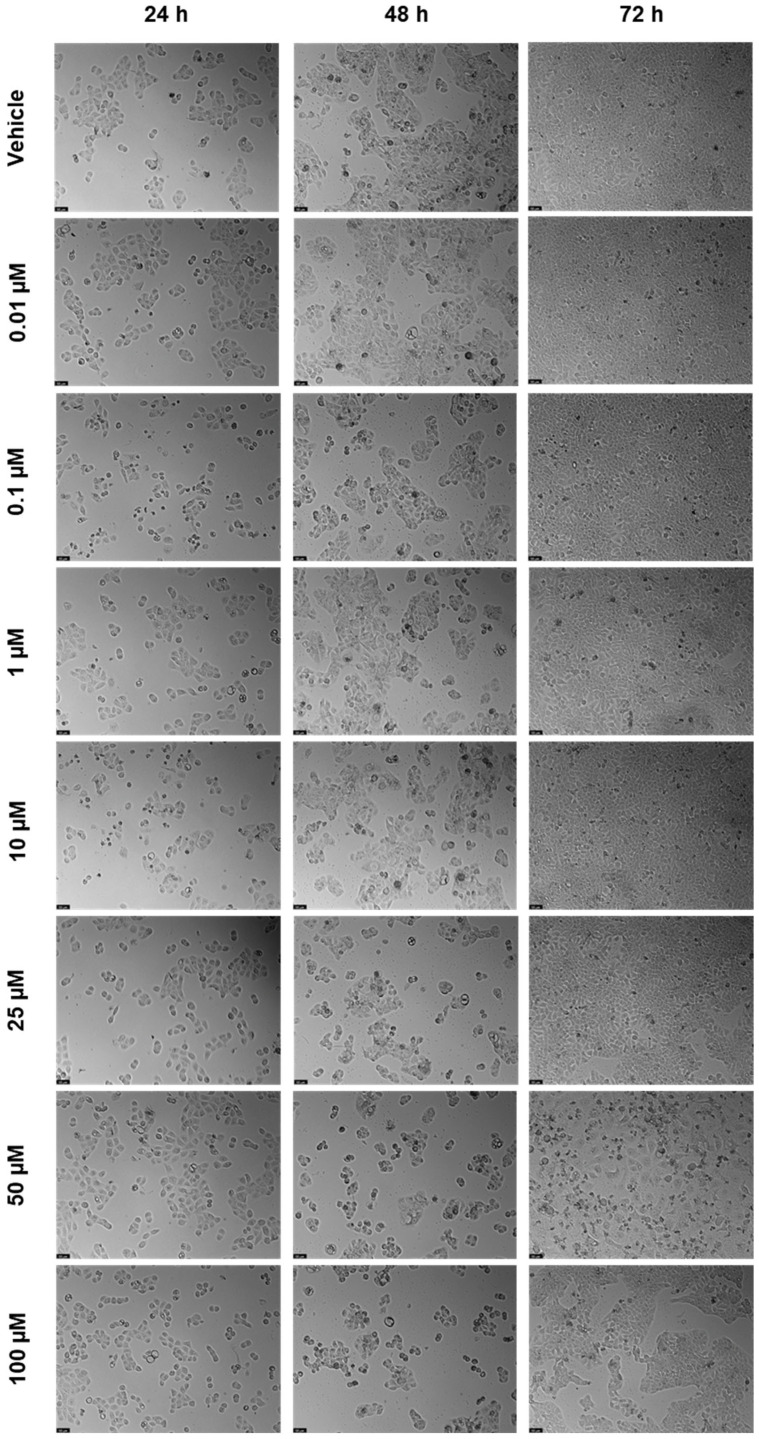
After being exposed to DRV at escalating concentrations (0.01–100 µM) for 24, 48, and 72 h, UM-UC-5 cell morphology was evaluated (*n* = 3). Negative control cells received the vehicle treatment (0.1% DMSO). The scale bar is 200 µm.

DRV was tested in the UM-UC-5 squamous bladder cancer cells at doses of 0.01, 0.1, 1, 10, 25, 50, and 100 µM at three time periods (24 h, 48 h, and 72 h) as one of the antivirals used in this investigation. Figure 2 and Figure 3 show, respectively, the outcomes of the morphological examination and cell viability testing. DRV had no significative effect for any concentration for 24 and 48 h (Figure 2a,b). At 72 h, there was a significant difference between the negative control and the two highest concentrations (50 and 100 µM), with 50 µM being the concentration with the strongest effect (Figure 2c). This can be confirmed by the morphological images, where the cells of 50 µM for 72 h are dispersed and with a lot of cell content outside the cells (Figure 3).

**Figure 4:**
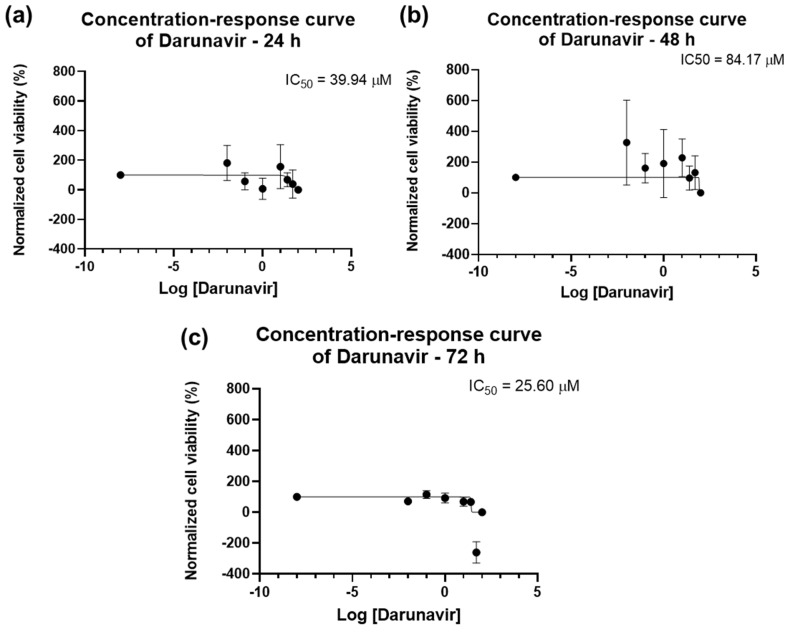
Dose-response curve and IC_50_ of UM-UC-5 following exposure to DRV at increasing concentrations for 24 h (**a**), 48 h (**b**), and 72 h (**c**) (concentrations of 0.01–100 µM). A 0.1% DMSO was applied to negative control cells (vehicle). Using the MTT assay, cell viability was determined. The findings were normalized and are presented as the mean ± SEM (*n* = 3).

The dose–response curves of DRV are represented in Figure 4. The program was able to calculate an IC_50_ for all time points: 39.94 µM for 24 h, 84.17 µM for 48 h, and 25.60 µM for 72 h. However, the value given for 24 h (39.94 µM, Figure 4a) does not match the results obtained in the graph bars of Figure 2, and as such is not considered reliable and, in this work, it is concluded that an IC_50_ for DRV at 24 h was not obtained. The IC_50_ of DRV decreased from 48 h to 72 h (Figure 4b,c), which indicates a time-dependent effect of DRV in bladder cancer cells.

#### 2.1.2. Cytotoxicity of Rilpivirine

**Figure 5:**
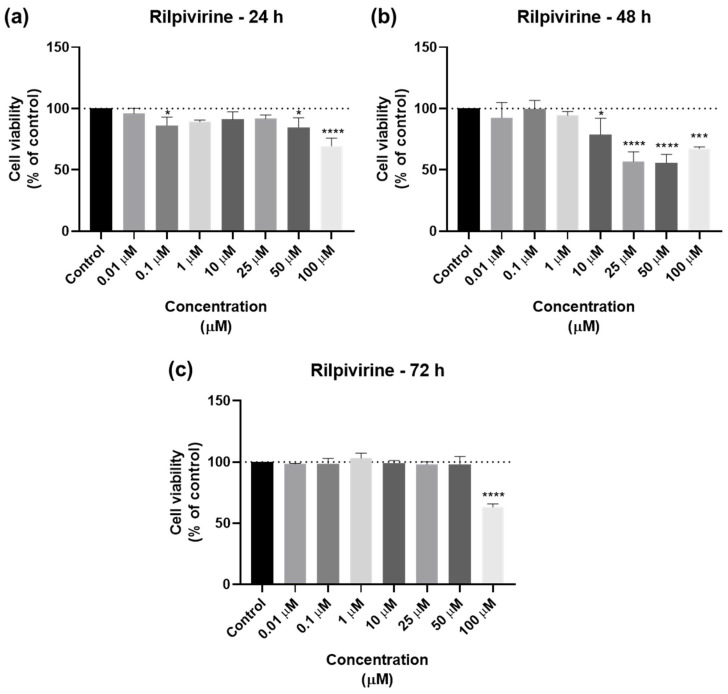
Results of UM-UC-5 cell viability following exposure to RPV at escalating doses (0.01–100 µM) for 24 h (**a**), 48 h (**b**), and 72 h (**c**). A 0.1% DMSO was applied to negative control cells (vehicle). The MTT assay was used to determine cell viability, and the findings are shown as the mean ± SEM (*n* = 3). * Statistically significant vs. negative control (vehicle) at *p* < 0.05; *** statistically significant vs. negative control (vehicle) at *p* < 0.001; **** statistically significant vs. negative control (vehicle) at *p* < 0.0001.

**Figure 6:**
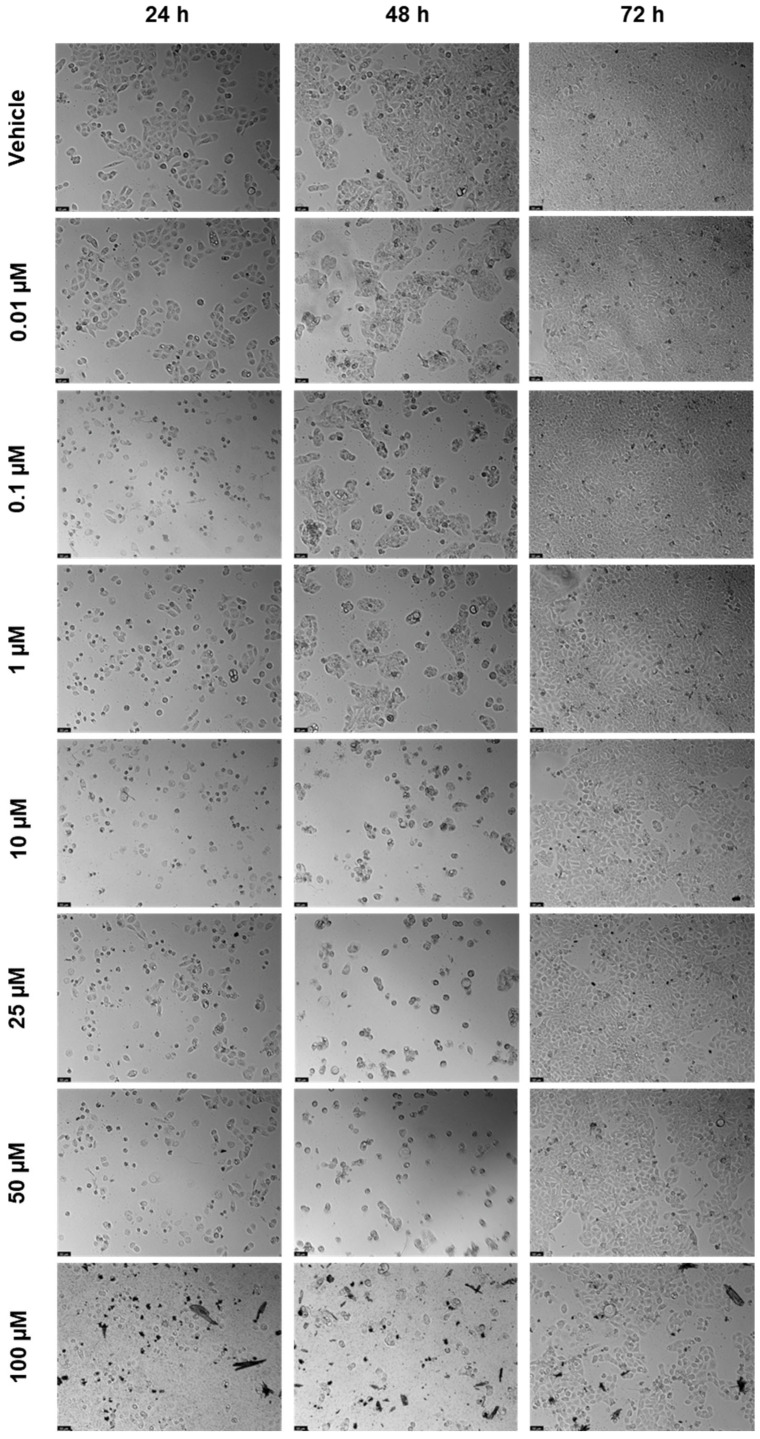
After being exposed to RPV at escalating concentrations (0.01–100 µM) for 24, 48, and 72 h, UM-UC-5 cell morphology was evaluated (*n* = 3). Negative control cells received the vehicle treatment (0.1% DMSO). The scale bar is 200 µm.

The cell viability results of UM-UC-5 cells exposed to RPV, and the corresponding cell morphology, are presented in Figure 5 and Figure 6. At 24 h, RPV was slightly effective at 50 and 100 µM (Figure 5a), while at 72 h, only 100 µM had a significative effect (Figure 5b). RPV was most effective in decreasing cell viability at 48 h in low concentrations of 10 µM. However, the effects of concentrations between 25–100 µM were similar among themselves (Figure 5c), which can also be seen in the microscopic images (Figure 6).

**Figure 7:**
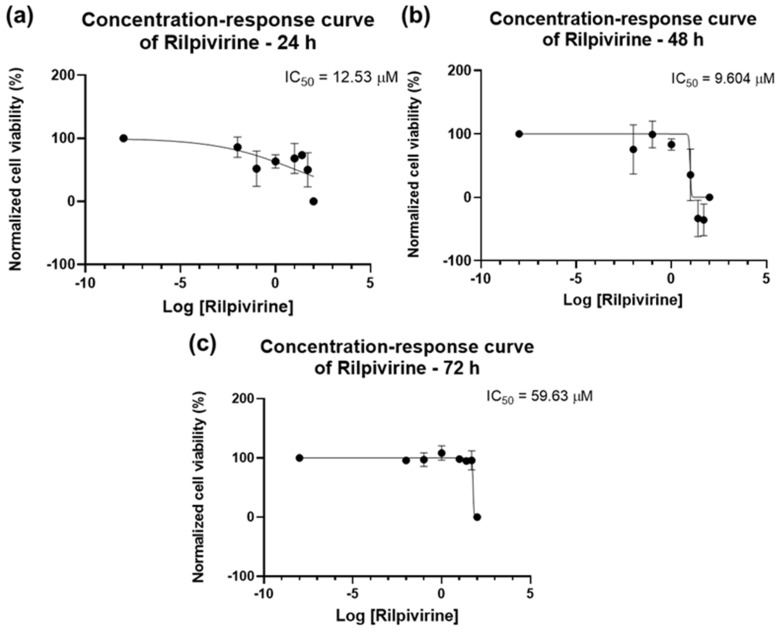
Dose–response curve and IC_50_ of UM-UC-5 following exposure to RPV at increasing concentrations for 24 h (**a**), 48 h (**b**), and 72 h (**c**) (concentrations of 0.01–100 µM). A 0.1% DMSO was applied to negative control cells (vehicle). Using the MTT assay, cell viability was determined. The findings were normalized and are presented as the mean ± SEM (*n* = 3).

The concentration–response curves for RPV are seen in Figure 7. An IC_50_ value of 12.53 µM for 24 h was obtained (Figure 7a), but, considering the bar graphs of Figure 5, this value was discarded. For 48 h, the IC_50_ was 9.604 µM (Figure 7b) and for 72 h, it was 59.63 µM (Figure 7c), which is per the results obtained above. Overall, RPV has the best results when used for 48 h.

#### 2.1.3. Cytotoxicity of Etravirine

**Figure 8:**
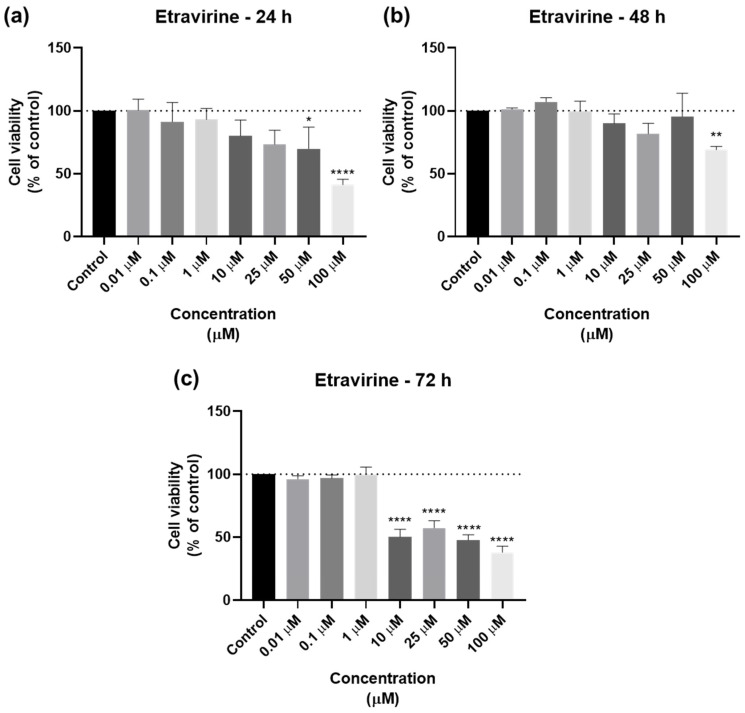
Results of UM-UC-5 cell viability following exposure to ETV at escalating doses (0.01–100 µM) for 24 h (**a**), 48 h (**b**), and 72 h (**c**). A 0.1% DMSO was applied to negative control cells (vehicle). The MTT assay was used to determine cell viability, and the findings are shown as the mean ± SEM (*n* = 3). * Statistically significant vs. negative control (vehicle) at *p* < 0.05; ** statistically significant vs. negative control (vehicle) at *p* < 0.01; **** statistically significant vs. negative control (vehicle) at *p* < 0.0001.

**Figure 9:**
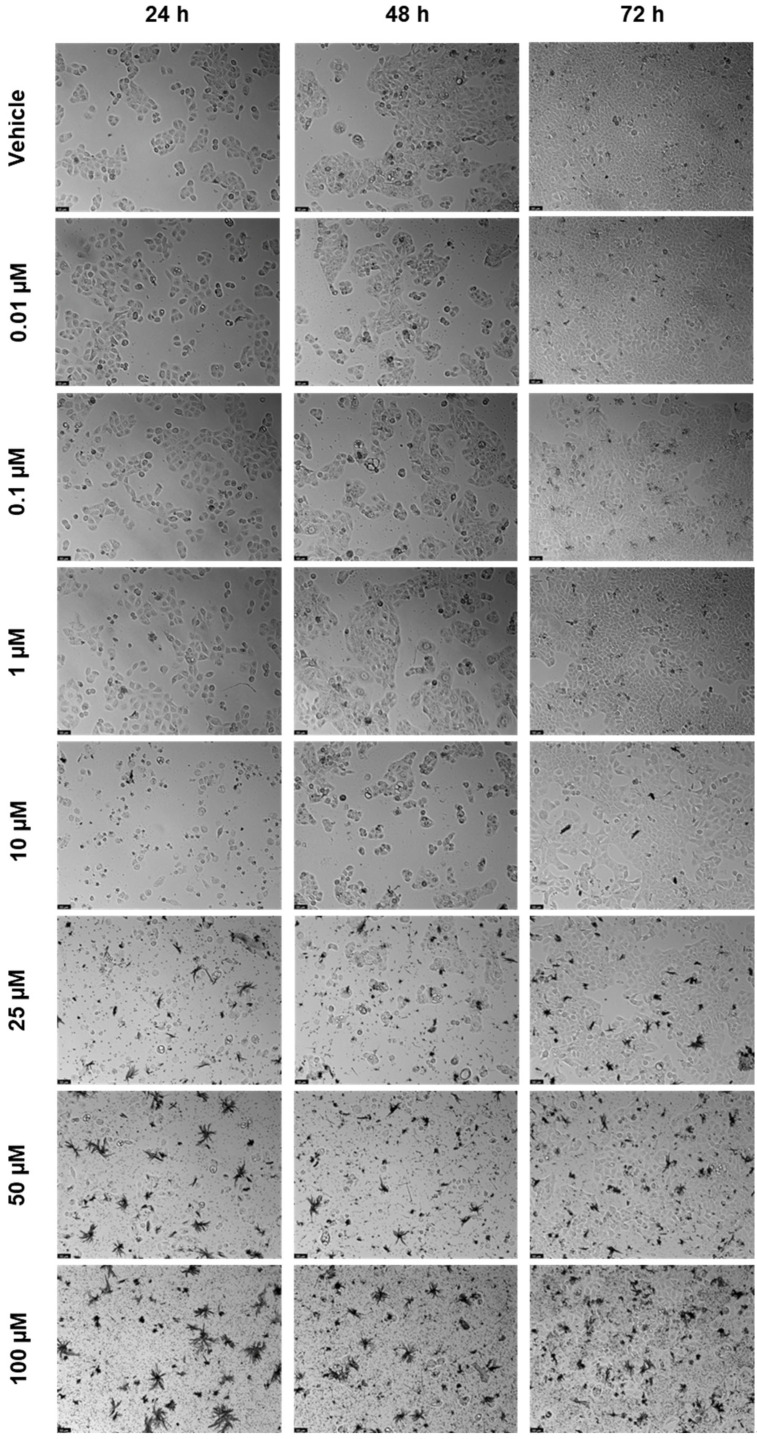
After being exposed to ETV at escalating concentrations (0.01–100 µM) for 24, 48, and 72 h, UM-UC-5 cell morphology was evaluated (*n* = 3). Negative control cells received the vehicle treatment (0.1% DMSO). The scale bar is 200 µm.

For ETV, the bar graphs and microscopic images are represented in Figure 8 and Figure 9. ETV reduced cell viability at all time points, but most notably at 72 h, when concentrations of 10 µM were already effective (Figure 8c). At 24 and 48 h, RPV’s effect was similar except for 50 µM, which had a significative difference from the negative control (Figure 8a,b). The microscopic images show the decrease, with the alteration of cell morphology accompanying this, as well as black fragments of ETV (Figure 9).

**Figure 10:**
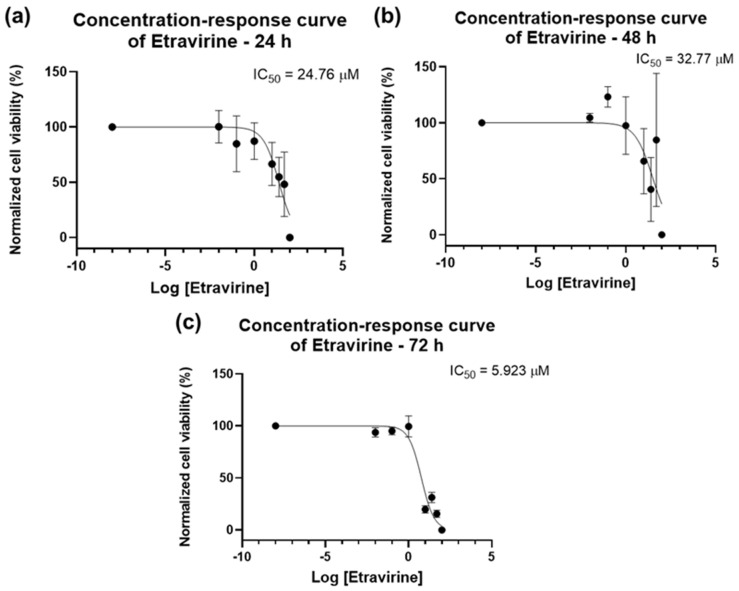
Dose–response curve and IC50 of UM-UC-5 following exposure to ETV at increasing concentrations for 24 h (**a**), 48 h (**b**), and 72 h (**c**) (concentrations of 0.01–100 µM). A 0.1% DMSO was applied to negative control cells (vehicle). Using the MTT assay, cell viability was determined. The findings were normalized and are presented as the mean ± SEM (*n* = 3).

The results of the bar graphs are reflected in the concentration–response curves of Figure 10. The IC_50_ of ETV for 24 h is slightly lower than that for 48 h (24.76 µM vs. 32.77 µM, Figure 10a,b), with 72 h having a lower IC_50_ of 5.923 µM (Figure 10c). The overall results show that ETV has a concentration-dependent effect in bladder cancer cells that is most effective for a longer time.

The IC_50_ values for all drugs and time points are summarized in Table 1. Only ETV had a trustable value at 24 h. DRV and ETV had a time-dependent effect, but ETV had similar effects between 24 h and 48 h, but these were slightly lower for 24 h. RPV had the best effect for 48 h. The drug that had the best overall effect on UM-UC-5 cells was ETV, while DRV had the least effect.

### 2.2. Drug Combinations

#### 2.2.1. Combination of Darunavir and Rilpivirine

**Figure 11:**
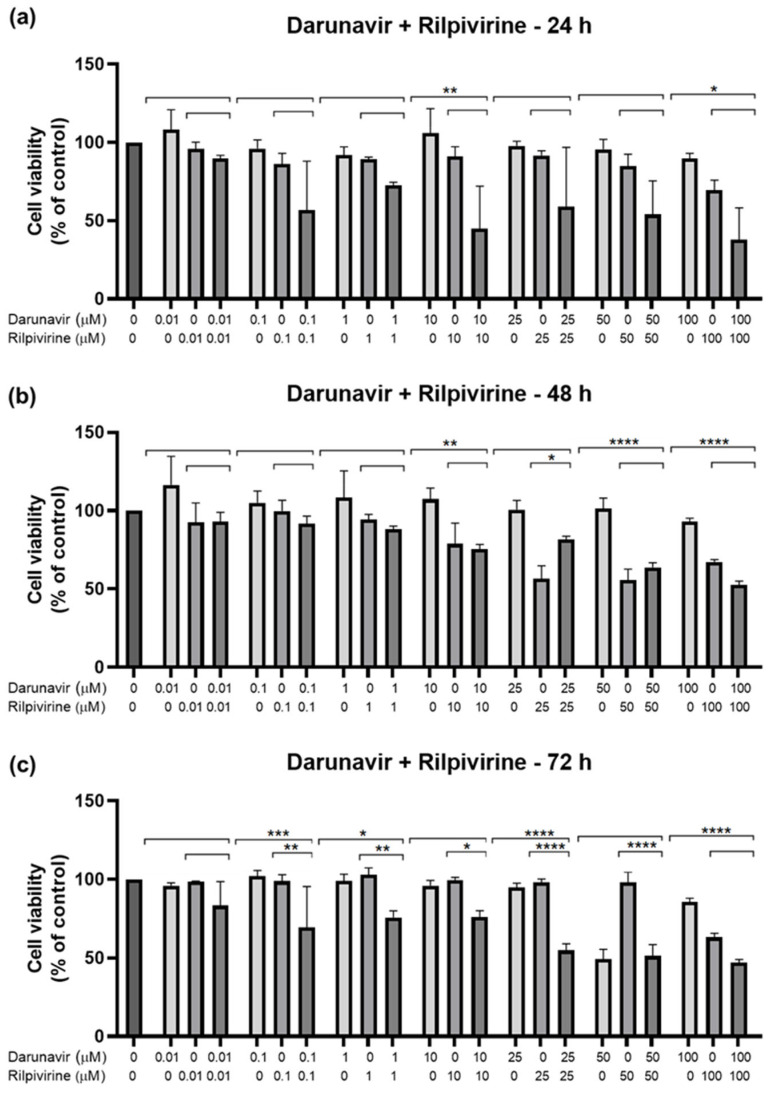
Results of UM-UC-5 cell cytotoxicity following exposure to single drugs and a combination of DRV and RPV for 24 h (**a**), 48 h (**b**), and 72 h (**c**). Both drugs were added at the same time. A 0.1% DMSO was applied to negative control cells (vehicle). The MTT assay was used to determine cell viability, and the findings are shown as the mean ± SEM (*n* = 3). * Statistically significant vs. drug alone at *p* < 0.05; ** statistically significant vs. drug alone at *p* < 0.01; *** statistically significant vs. drug alone at *p* < 0.001; **** statistically significant vs. drug alone at *p* < 0.0001.

**Figure 12:**
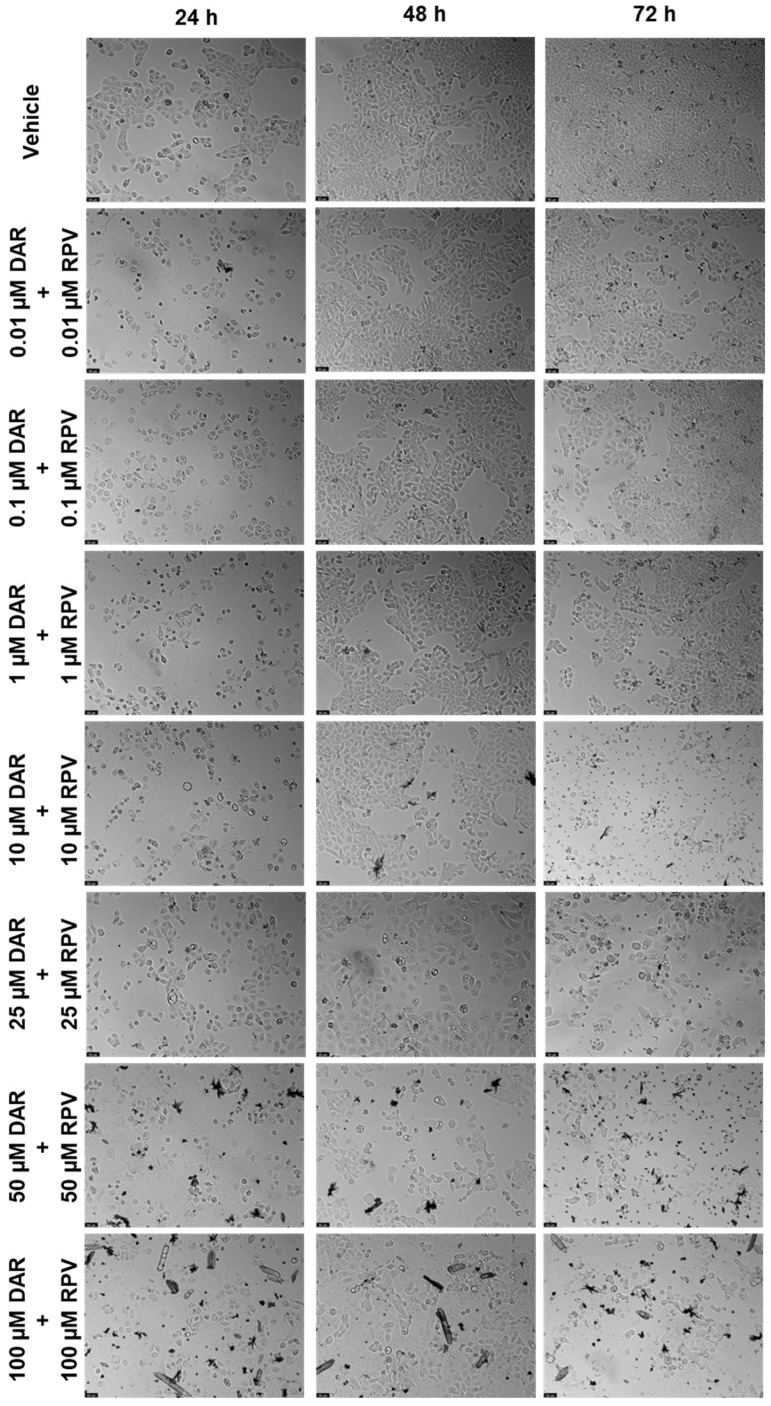
Morphological evaluation of UM-UC-5 cells after exposure to combinations of DRV and RPV at increasing concentrations for 24 h, 48 h, and 72 h. Both drugs were added at the same time. Negative control cells were treated with the vehicle (0.1% DMSO). These images are representative of three independent experiments. The scale bar is 200 μm.

After testing the drugs alone, a study of the combination between them was performed to assess if the drugs acted better alone or with each other. The method of this combination study was to test the same concentrations of each drug for the three time points, and the first pair was DRV and RPV. Figure 11 shows the cell viability bar graphs, where the combination cell viability is compared with each drug alone, and Figure 12 shows the morphological analysis. The combination of these drugs was only significatively more effective than both drugs alone for 72 h, at concentrations of 0.1, 1, and 25 µM (Figure 11c). For the other concentrations and time points, the combination of DRV and RPV was more effective than only one of the drugs alone, which means that the effect can be attributed to one of the drugs alone (Figure 11a,b). Values of the cell viability percentage scarcely dropped below 50%, with the 10 µM and 100 µM of DRV and RPV at 24 h being some of the lowest.

#### 2.2.2. Combination of Etravirine and Rilpivirine

**Figure 13:**
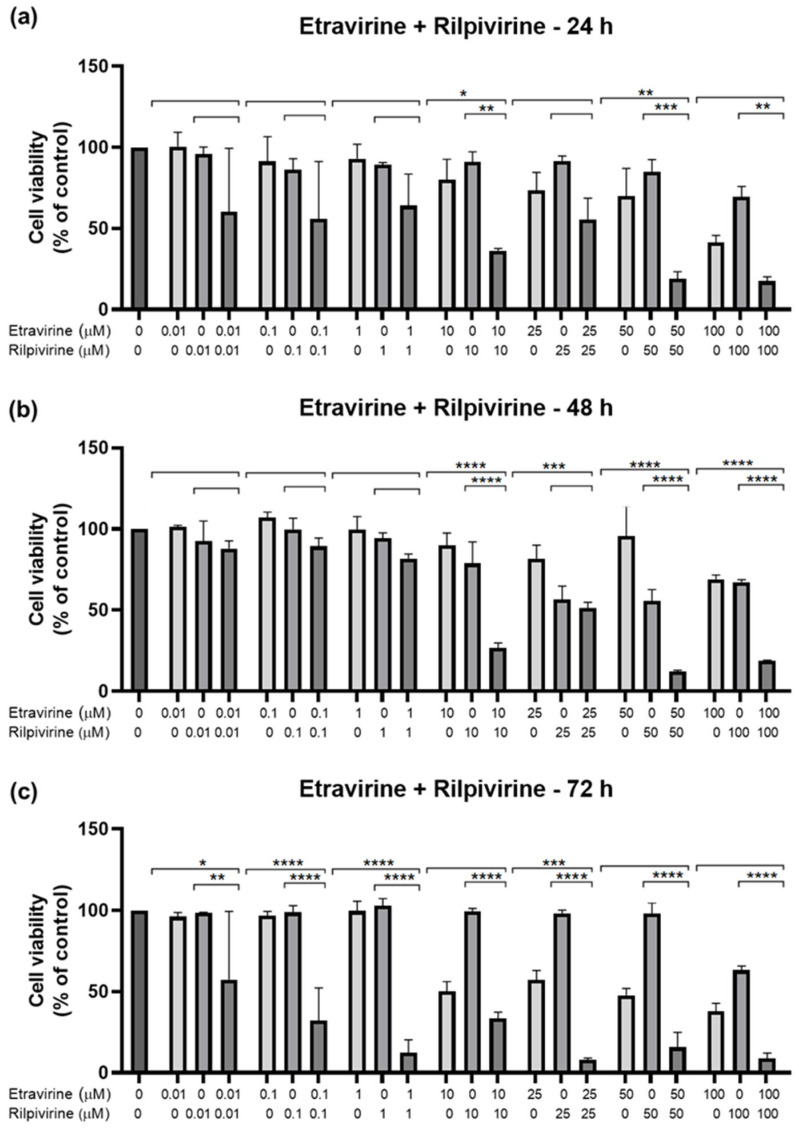
Results of UM-UC-5 cell cytotoxicity following exposure to single drugs and a combination of ETV and RPV for 24 h (**a**), 48 h (**b**), and 72 h (**c**). A 0.1% DMSO was applied to negative control cells (vehicle). The MTT assay was used to determine cell viability, and the findings are shown as the mean ± SEM (*n* = 3). * Statistically significant vs. drug alone at *p* < 0.05; ** statistically significant vs. drug alone at *p* < 0.01; *** statistically significant vs. drug alone at *p* < 0.001; **** statistically significant vs. drug alone at *p* < 0.0001.

**Figure 14:**
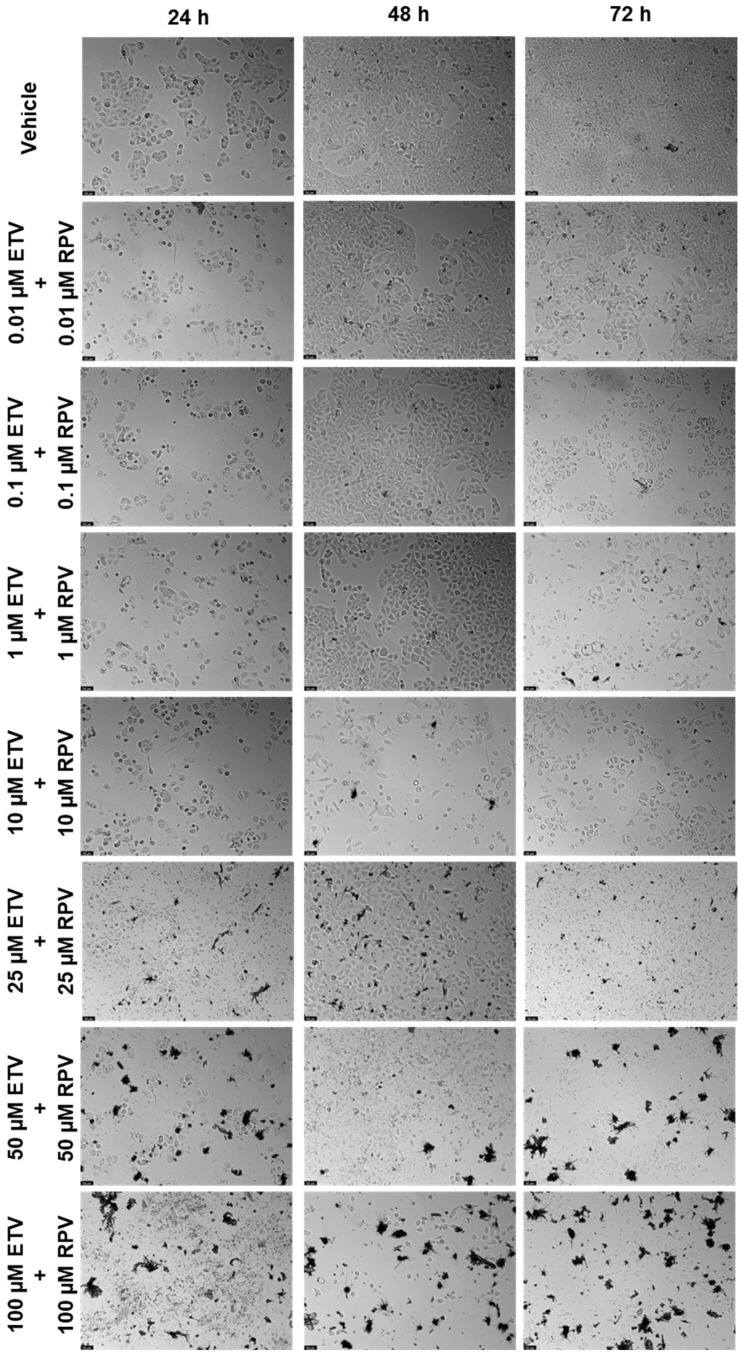
Morphological evaluation of UM-UC-5 cells after exposure to combinations of ETV and RPV at increasing concentrations for 24 h, 48 h, and 72 h. Both drugs were added at the same time. Negative control cells were treated with the vehicle (0.1% DMSO). These images are representative of three independent experiments. The scale bar is 200 μm.

The next combination tested was ETV and RPV, and the results are represented in Figure 13 and Figure 14. The combinations caused a concentration and time-dependent decrease in cell viability. For 24 h, the combinations that were better than both drugs alone were 10 and 50 µM (Figure 13a), for 48 h, the concentrations were 10, 50, and 100 µM (Figure 13b), and for 72 h, the concentrations were 0.01, 0.1, 1, and 25 µM. Visually, the combinations cause cell viability to a significative decrease in a concentration and time-dependent manner, with cell viability being below 50% beyond 10 µM for 24 h and 48 h, and already at 0.1 µM of each drug for 72 h. The cell morphology images show a decrease in cell density, accompanied by an altered morphology due to cell death (Figure 14).

#### 2.2.3. Combination of Etravirine and Darunavir

**Figure 15:**
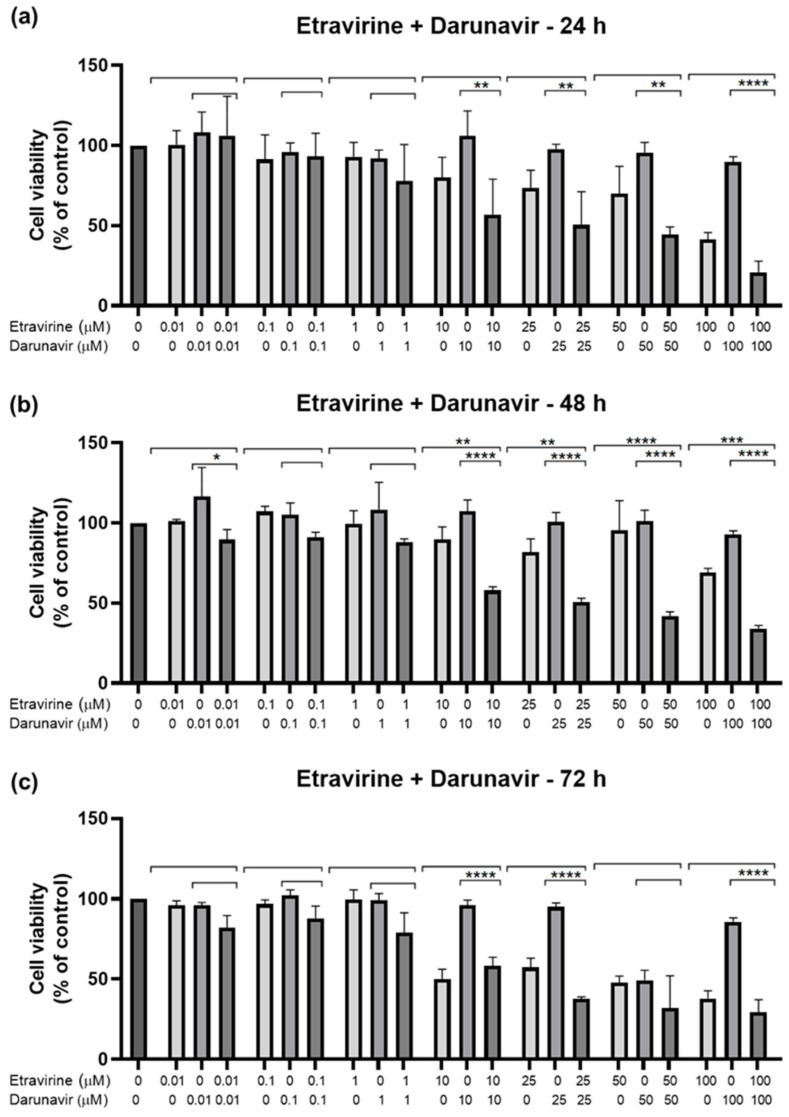
Results of UM-UC-5 cell cytotoxicity following exposure to single drugs and a combination of ETV and DRV for 24 h (**a**), 48 h (**b**), and 72 h (**c**). A 0.1% DMSO was applied to negative control cells (vehicle). The MTT assay was used to determine cell viability, and the findings are shown as the mean ± SEM (*n* = 3). * Statistically significant vs. drug alone at *p* < 0.05; ** statistically significant vs. drug alone at *p* < 0.01; *** statistically significant vs. drug alone at *p* < 0.001; **** statistically significant vs. drug alone at *p* < 0.0001.

**Figure 16:**
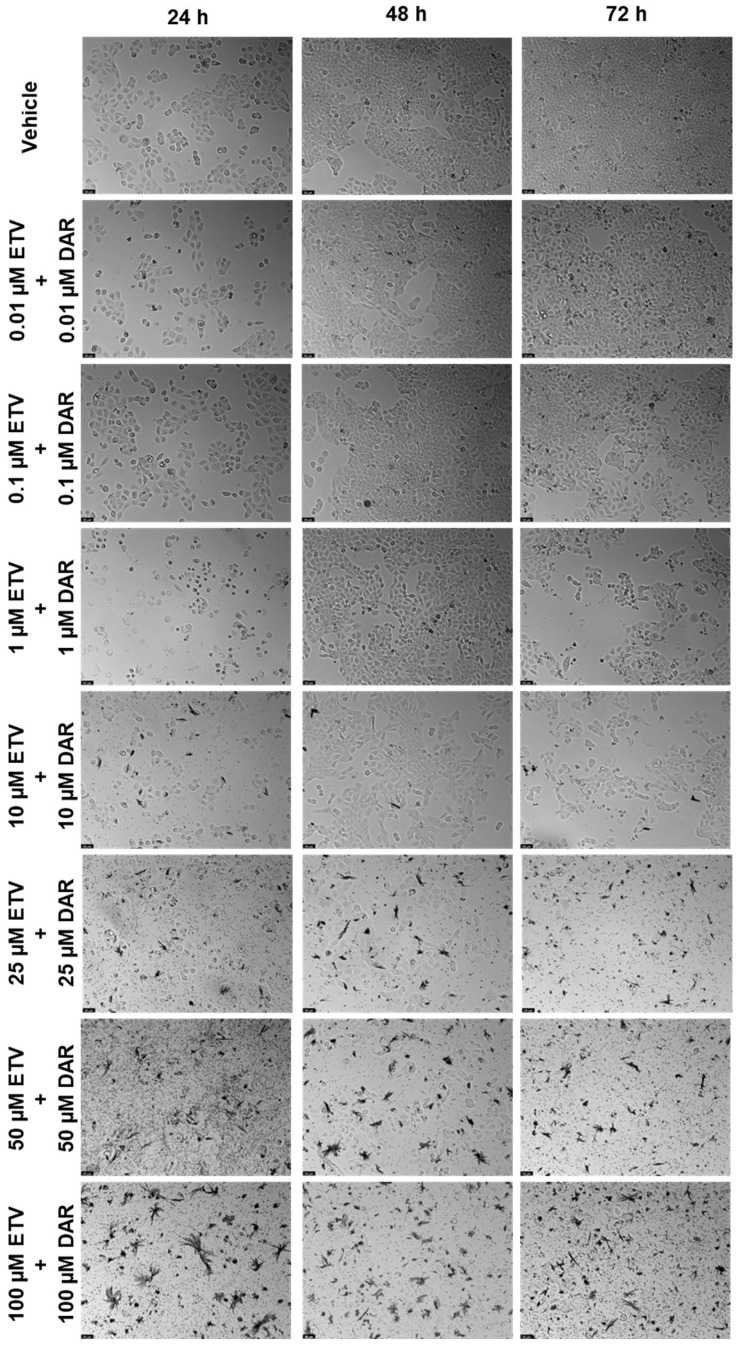
Morphological evaluation of UM-UC-5 cells after exposure to combinations of ETV and DRV at increasing concentrations for 24 h, 48 h, and 72 h. Both drugs were added at the same time. Negative control cells were treated with the vehicle (0.1% DMSO). These images are representative of three independent experiments. The scale bar is 200 μM.

The last concentration was ETV and DRV (Figure 15 and Figure 16). This combination’s effect was concentration-dependent, but across the different time points, the decrease in viability is similar for each concentration combination. The time point where the combination was greater than each drug alone was 48 h, for 10, 25, 50, and 100 µM (Figure 15b). Cell viability’s lowest decrease by combination was at 48 h, which was below 50% beyond 10 µM of each drug. At other times there was also a decrease, but it can be attributed primarily to ETV activity. Again we can note decreased cell density with increasing concentrations, as well as an accumulation of darker colored spots, that can either be small fragments of drugs or intracellular content of dead cells (Figure 16).

These results show that the best combination is ETV and RPV, which caused the greatest decrease in cell viability of all combinations, at higher concentrations for 24 h (10 and 50 µM) and 48 h (10–100 µM), and in lower ones for 72 h (0.01–25 µM). The second-best combination is ETV with DRV for 48 h with a concentration of 10–100 µM each. Lastly, the worst combination was RPV and DRV, which was only significatively different from both drugs alone at 72 h but had a small decrease in cell viability when compared with the other combinations.

### 2.3. DECREASE Matrices

**Figure 17:**
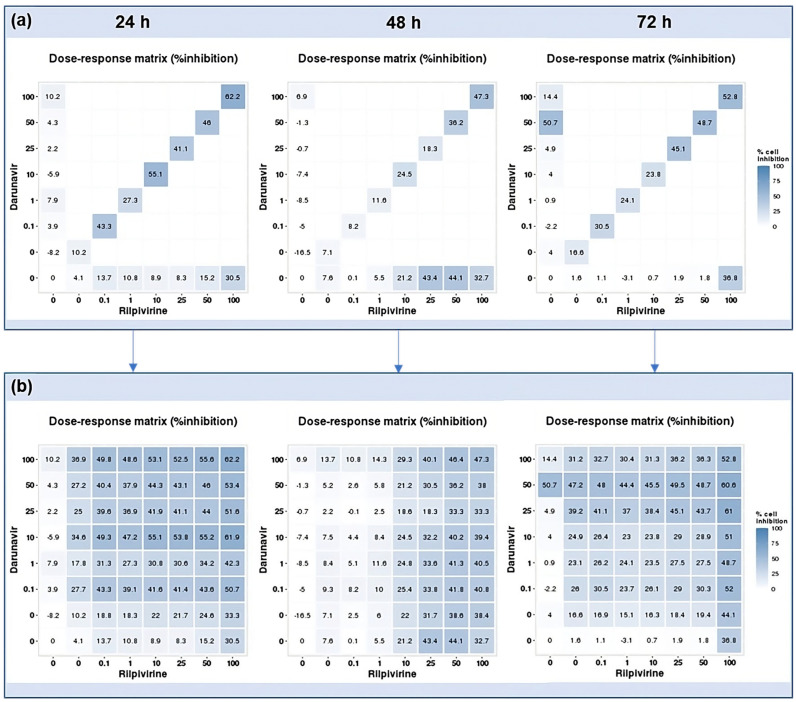
DECREASE output. The cell inhibition values of DRV alone (first column), RPV alone (bottom row), and in combination at the same concentrations (diagonal) were input into the DECREASE web application to form the incomplete dose–response matrices for 24, 48, and 72 h (**a**). The predicted full matrices of all combinations for the three time points were then generated by DECREASE using the Non-negative Matrix Factorization cNMF algorithm (**b**).

**Figure 18:**
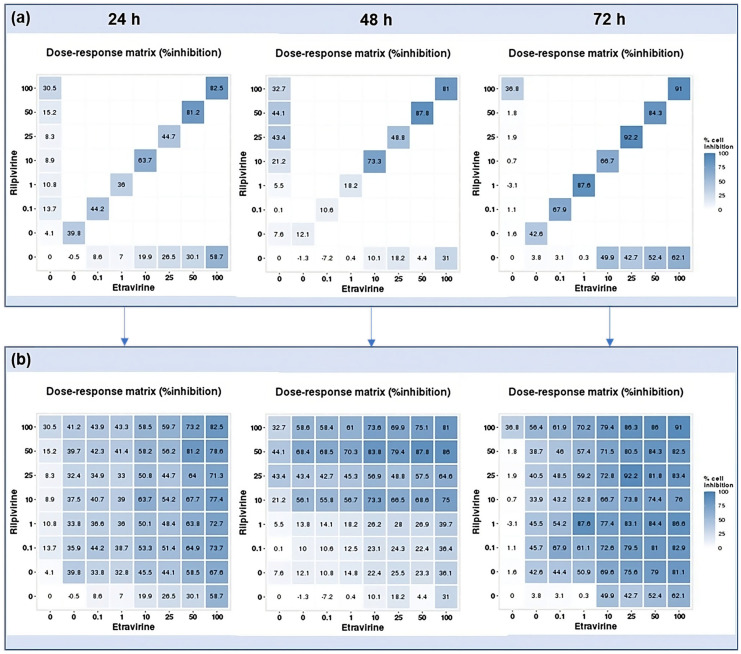
DECREASE output. The cell inhibition values of RPV alone (first column), ETV alone (bottom row), and in combination at the same concentrations (diagonal) were input into the DECREASE web application to form the incomplete dose–response matrices for 24, 48, and 72 h (**a**). The predicted full matrices of all combinations for the three time points were then generated by DECREASE using the Non-negative Matrix Factorization cNMF algorithm (**b**).

**Figure 19:**
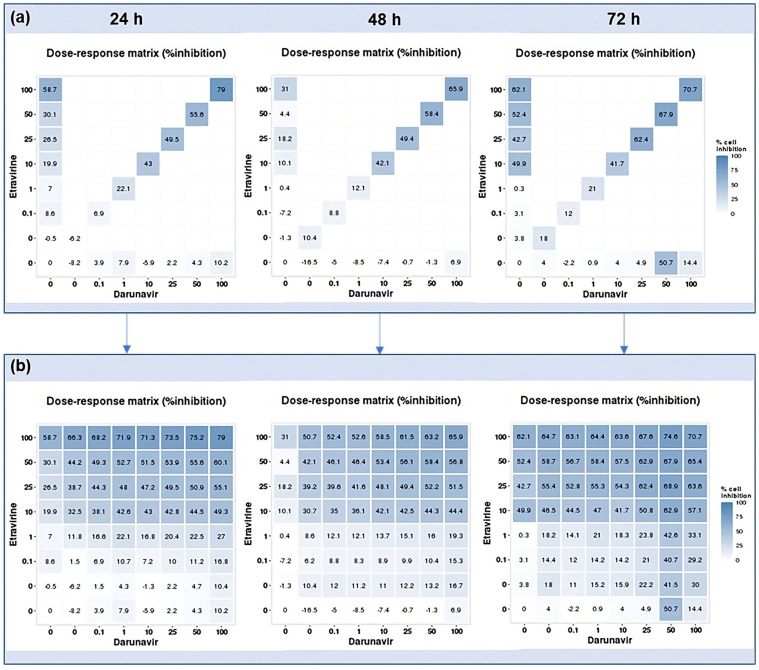
DECREASE output. The cell inhibition values of ETV alone (first column), DRV alone (bottom row), and in combination at the same concentrations (diagonal) were input into the DECREASE web application to form the incomplete dose–response matrices for 24, 48, and 72 h (**a**). The predicted full matrices of all combinations for the three time points were then generated by DECREASE using the Non-negative Matrix Factorization cNMF algorithm (**b**).

Using the reverse of the cell viability results obtained experimentally, the dose–response cell inhibition incomplete matrices of the combinations were established, and the DECREASE software was used to predict the complete matrices. Figure 17 demonstrates these results for the combination of DRV and RPV for all time points studied, Figure 18, the ETV and RPV combinations, and Figure 19, the matrices for DRV and ETV. Overall, the matrices for ETV and RPV have higher cell inhibition percentages, as expected when considering the in vitro combination results, followed by DRV and RPV, but with higher concentrations. Some preliminary studies were made to assess if the predicted cell inhibition percentages could be reproduced experimentally and, especially for DRV and RPV at 48 h, the results are promising, and this will be further explored in future works.

### 2.4. SynergyFinder Results

#### 2.4.1. Darunavir and Rilpivirine

With the full matrices predicted by the DECREASE web application, an in silico study of the synergy of the pairwise drug combinations was carried out using SynergyFinder. The results were obtained in the form of heat maps, where a red color indicates synergism and a green color, antagonism, with the intensity of the color indicating the degree of the interaction. The Bliss–Loewe synergy scores give an idea of the overall combination synergism.

**Figure 20:**
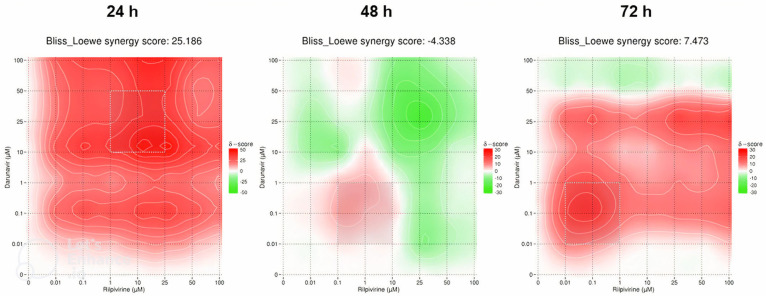
SynergyFinder scores and 2D synergy maps for the combination of DRV and RPV for 24, 48, and 72 h. The red areas indicate synergism, while the green areas indicate antagonism. Bliss–Loewe scores lower than −10 are indicative of the combination being antagonistic, between −10 and 10 are additive, and above 10, the combination is synergistic. The most synergistic areas (three-by-three concentration windows) for each time are highlighted.

The first combination was DRV and RPV and the heat maps are pictured in Figure 20. At 24 h, the Bliss–Loewe score was 25.186, which is indicative of synergism, with the most synergistic area (MSA) being for the concentrations of 10–50 µM of DRV and 1–25 µM of RPV. The color was overall red, with the greater synergism being for the concentrations 10 µM of DRV and 25 and 50 µM of RPV (Figure 20). At 48 h, the synergy score was −4.338, which is indicative of additivity, and the heat map shows an overall green color, markedly more intense for higher concentrations, which represents a more antagonistic response at those concentrations. The MSA is between 0.01–1 µM DRV and 0.1–10 µM RPV, but even then, the combinations are additive at best (Figure 20). At 72 h, the score of 7.473 also indicates an overall additive effect of the combination. However, the map demonstrates a red color, with the MSA being in the concentrations of 0.01–1 µM of DRV and RPV, and the most synergy concentrations being at 0.1 µM of DRV and RPV, and 25 µM of DRV with 25–100 µM of RPV (Figure 20).

#### 2.4.2. Etravirine and Rilpivirine

**Figure 21:**
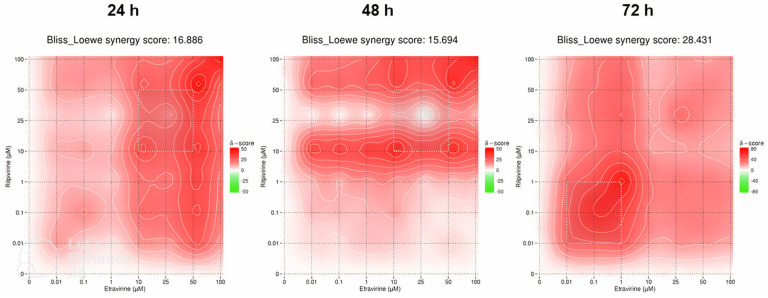
SynergyFinder scores and 2D synergy maps for the combination of RPV and ETV for 24, 48, and 72 h. The red areas indicate synergism, while the green areas indicate antagonism. Bliss–Loewe scores lower than −10 are indicative of the combination being antagonistic, between −10 and 10 are additive, and above 10, the combination is synergistic. The most synergistic areas (three-by-three concentration windows) for each time are highlighted.

The synergy heat maps in Figure 21 are of the ETV and RPV combinations. At 24 h of cell exposure, the Bliss–Loewe score was 16.886, which indicates a synergistic relationship, with the MSA being between the concentrations of 10–50 µM of ETV and RPV. The map shows a tendency for the red synergistic color with an increase of concentrations, with a more pronounced shift when the ETV concentration is increased (Figure 21). At 48 h, the Bliss–Loewe score is 16.694, similar to the 24 h score and also synergistic, and the MSA is also the same. At this time point, however, the RPV concentration is more influent in the synergy, with redder areas along the 1–25 µM and 50–100 µM ranges of RPV, for all concentrations of ETV (Figure 21). Lastly, the 72 h time point had the best synergy score, at 28.431 and the lowest MSA concentrations of 0.01–1 µM of ETV and RPV. The map shows an overall red color with lower concentrations being more synergistic (Figure 21).

#### 2.4.3. Etravirine and Darunavir

**Figure 22:**
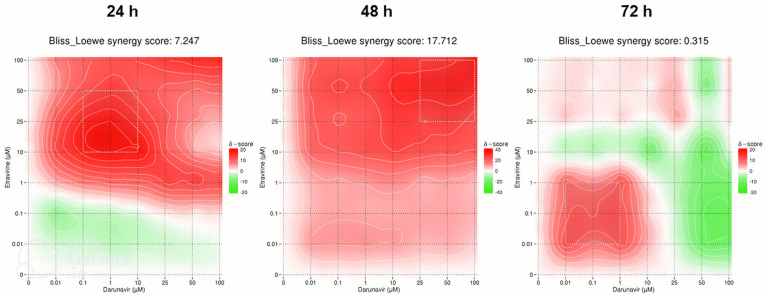
SynergyFinder scores and 2D synergy maps for the combination of ETV and DRV for 24, 48, and 72 h. The red areas indicate synergism, while the green areas indicate antagonism. Bliss–Loewe scores lower than −10 are indicative of the combination being antagonistic, between −10 and 10 are additive, and above 10, the combination is synergistic. The most synergistic areas (three-by-three concentration windows) for each time are highlighted.

The last synergy heatmaps show the combination of ETV and DRV and are represented in Figure 22. The overall Bliss–Loewe score for 24 h is 7.247, which is an additivity value. The map demonstrates a green color for lower concentrations of both drugs, which shows an antagonistic relationship, but beyond 1 µM of ETV, the map becomes synergistic red, with the MSA in the concentrations of 0.1–10 µM of DRV and 10–50 µM of ETV (Figure 22). At 48 h, the synergistic score is higher at 17.712 and is synergistic, with the overall red color of the map also being consistent with that interpretation. The synergism is more pronounced with the increase of ETV, and the MSA is in the concentrations of 25–100 µM of both drugs (Figure 22). The final Bliss–Loewe score of this study is for 72 h and is 0.315, with the additive result. The map shows a green color for high concentrations of DRV, with the red color being more present at lower concentrations of both drugs, while the MSA is also indicated at concentrations between 0.01–1 µM of DRV and ETV. This demonstrates that high concentrations of DRV have an antagonistic effect on cell inhibition (Figure 22).

A summary table of the synergy scores has been made to facilitate comprehension (Table 2). Overall, the most synergistic specific combinations are DRV + RPV at 24 h and ETV + RPV at 72 h, with ETV + RPV being the only combination that was synergistic for all time points. The other combinations are only synergic at one time point, with DRV + RPV at 24 h and ETV + DRV at 48 h.

The specific Bliss–Loewe synergistic scores of the MSAs are also indicated. This helps to understand that for the additive overall combinations of DRV + RPV at 72 h and ETV + DRV at 24 h, the MSA is synergistic, while for the additive combinations of DRV + RPV at 48 h, the MSA is additive. The MSA for ETV + DRV at 72 h is bordering on synergism. The MSAs with the highest scores are for DRV + RPV at 24 h, ETV + RPV for all concentrations, and ETV + DRV at 48 h, with the absolute best being DRV + RPV at 24 h and ETV + RPV at 72 h, which is consistent with the higher synergy combinations.

Overall, the best and most synergic combination is ETV + RPV, especially at 72 h.

## 3. Discussion

Drug repurposing is an emerging technique for the development of new cancer treatments. This offers several advantages, such that the medicine has already been demonstrated to be safe for use in humans, the drug development process is sped up, and it also costs less [20]. This approach has already been used in recent years, and up to 30% of drugs approved by the FDA have been repurposed drugs [21]. In oncology specifically, there have been several drugs studied from other diseases to be applied in cancer treatment, such as raloxifene and celebrex, both of which are for osteoporosis and have been repurposed to breast and colon cancer, respectively [22,23], but also for antineoplastic drugs being applied to other diseases, such as tamoxifen, which is used in metastatic breast cancer and has been repurposed as an antimicrobial and for bipolar disorder [24,25]. The idea is typically to search for drugs that can also act on specific cancer therapy targets, such as epidermal growth factor receptors, cyclin-dependent kinases, Ras protein, and cancer stem cells [26]. These kinds of repurposing studies have been performed using in silico methods such as molecular docking and machine learning [27,28], but posterior validation using pre-clinical and clinical studies is always done [29].

In this study, DRV, RPV, and ETV, three antiretroviral drugs, were studied in UM-UC-5 bladder cancer cells. These drugs have been selected because they have been shown to have effects on cancer in studies found in the literature, namely ETV in ovarian cancer metastasis [30], RPV in pancreatic cancer [31], and DRV has demonstrated in silico evidence of inhibiting enzymes overexpressed in several cancer types [32]. Overall, the best drug tested in this study was ETV, in a concentration and time-dependent manner, having a relatively low IC_50_ at 24 and 48 h (24.76 µM and 32.77 µM), but with the overall lowest IC_50_ being at 72 h (5.923 µM). This is following the literature found, since ETV has been the most studied drug among these three. A potential mode of action that has been explored is the inhibition of casein kinase 1 ε (CK1ε), which is an enzyme included in a family of enzymes that are involved in signal transduction pathways [33]. This enzyme is a positive regulator of the WNT/β-catenin pathway, activated by WNT and responsible for the phosphorylation of the Dishevelled protein that ensures the stability of β-catenin by inhibiting its degradation complex [34]. The dysregulation of these pathways is associated with the development of early carcinogenesis, with an accumulation of β-catenin in the nucleus which induces cell proliferation [35]. The inhibition of CK1ε has, therefore, been explored and has demonstrated results in inducing cell cycle arrest and apoptosis in cancer cells, and ETV has been proposed as an inhibitor of this molecule. This drug was selected among the FDA-approved drug library in a virtual screening as being highly capable of binding to CK1ε, with similar results to the CK1ε umbralisib, showing the promise of ETV in inhibiting this enzyme [33], which could be a path that relates to the results in the present article. Another mode of action that could be behind ETV’s capacity to decrease the viability of bladder cancer cells is the inhibition of the human anterior gradient protein 2 homolog (AGR2). This protein is a disulfide isomerase expressed in the endoplasmic reticulum that regulates protein folding and is related to the initiation of carcinogenesis, its progression, and resistance to therapy [36], and is overexpressed in bladder cancer cells and can be related to the local spread of cancer, and the secretion of this protein by bladder cancer cells can be used as a biomarker [37]. ETV in ovarian cancer cells was able to decrease AGR2 levels, as well as induce autophagy by increasing a key component in its pathway (LC3-B), suppressing cell proliferation, migration, and invasion when used alone, in vitro and in vivo tumor growth and metastasis when used in combination with the antineoplastic drug paclitaxel [30]. The latter is likely due to the interaction of AGR2 secreted from cancer cells with VEGF and FGF2, a bond that eases angiogenesis and, consequently, metastasis [38]. The concentrations at which ETV showed results were 5–10 µM at different time points [30], which is slightly lower than the results obtained here for 24 and 48 h, but accordant to those obtained for 72 h, which can be attributed to different cancer cell types.

RPV can be considered the next best drug, markedly at 48 h (9.604 µM). This drug has been tested in several cancer cell types, but never in bladder cancer. Of note, a study that used 10 cancer cell lines, from breast, lung, cervical, and liver cancer, among others, showed that this drug is also effective at 48 h in most of them with an IC_50_ ranging from 4.3–87.4 µM, showing that the results obtained in this study are lower than most obtained in the literature for the same time point, only having a lower result in acute leukemia cells, demonstrating the extreme promise of RPV in bladder cancer [39]. In contrast, the IC_50_ obtained for 72 h (59.63 µM) was higher than what was obtained in two studies, one for acute myeloid leukemia and colorectal, pancreatic, and ovarian carcinoma [40], and the other for pancreatic cancer [31]. In the former, the IC_50_ ranged from 3.045–9.422 µM, and in the latter from 16.2–24.4 µM, with the only IC_50_ higher being 294 µM for a specific pancreatic cell line [31,40]. In terms of the mechanisms of action of RPV, several have been proposed. One of the studies mentioned above displayed an inhibitory effect of RPV on aurora A kinase [40], which is commonly overexpressed in cancer that induces entry into the cell cycle, the inactivation of DNA damage checkpoints, and the decrease of apoptosis [41]. This kinase of the serine/threonine family is amplified in invasive bladder cancer and is connected with poor prognosis, both due to its effects on mitosis and genomic instability but also due to its directly increasing the invasiveness of bladder cancer cells. It can also be used as a non-invasive biomarker present in urine, so the capability of RPV inhibiting aurora A kinase can be directly associated with the effect obtained in this cancer [42]. Another pathway explored was the vascular endothelial growth factors–receptors (VEGFs-VEGFRs) pathway, closely associated with angiogenesis in cancer and of which the VEGFR-2 receptor is a key player since its autophosphorylation activates downstream angiogenic pathways, as well as its promotion of cell survival and division [43]. RPV had an inhibitory effect of VEGFR-2 at an IC_50_ of around 5.45 µM, which is quite similar to the values obtained here [44]. This pathway and its constituents are increased in the bladder cancer tissues of patients, particularly VEGFR-2, and are also related to poor prognosis and recurrence [45], and, as such, the inhibition of this receptor can explain the cytotoxicity of RPV in bladder cancer cells observed in this study.

The worst drug tested in this study was DRV, only having a lower IC_50_ at 72 h (25.60 µM). This was expected since this drug was used in very few experiments on cancer cells in the literature, and when it was tested it yielded no effect. However, this could be related to the duration of the experiments since, for example, in a study that tested DRV in primary effusion lymphoma, only 24 h time points were used, and the present study demonstrated that this drug has no effect when only exposed for that time [46]. Presumably, if left for up to 72 h, DRV could have had a decrease in cell viability and division, as well as in the inhibition of nuclear factor kappa B (NF-κB), which was one of the parameters of the study. The activation of this factor is commonplace in cancer and causes an escape from apoptosis, leading to cell survival and metastatic dissemination. In bladder cancer, NF-κB upregulates survivin expression, which increases the cell cycle and resistance to apoptosis, promoting cancer progression and drug resistance [47]. Therefore, although the study showed no effect of DRV in NF-κB reduction, the assessment of if it is capable of doing so at 48 and 72 h is of interest for future research. More recently, an in silico study has demonstrated that DRV has a high affinity for binding to the active site of the human lactate dehydrogenase A (LDHA) enzyme, with high stability hydrogen bonds. This is similar to known inhibitors and the article concludes that DRV can be a potential LDHA inhibitor [32]. This enzyme converts pyruvate into lactate and is often upregulated in cancer, promoting several of the known hallmarks of cancer, such as increased proliferation, cell invasion and metastasis, angiogenesis, and immune escape, and its inhibition has been known to impair cancer progression [48]. Bladder cancer is no exception, as LDHA is increased in the cells and boosts glycolysis, proliferation, and invasion [49], and serum levels can be used as a biomarker and are associated with decreased overall and progression-free survival, particularly in non-urothelial carcinoma of the bladder cancers such as squamous bladder cancer [50].

Drug combination is widely used for the treatment of all diseases, and cancer is no exception, so in this article, the drugs were used in combination to understand if there were improved effects of these drugs when combined. Differently from previous studies of grouping where the IC_50_ is used for combination [51], the combination model used was combining drugs in the same concentrations. This change was made to use the DECREASE web tool, which allows for a high-throughput screening of drug combination by extrapolating a multi-dose–response cell inhibition matrix from the values of cell inhibition alone and in a diagonal, same concentration, pairwise combination. This allows the identification of potentially effective drug combinations without having to test the whole array of combinations, and also the use of synergy calculation software, as was done in the present article using SynergyFinder [52]. The best combination in this study was ETV with RPV, which was expected since these drugs had the best effects alone as well, with the best effects recorded at 48 and especially at 72 h, with those at 72 h already at low concentrations of 0.1 µM each. This is also mirrored in the synergy score since this combination has the highest Bliss–Loewe synergy score overall, and in the MSA area. These two drugs are similar, as they are both second-generation diarylpyrimidine NNRTIs, which can contribute to their high synergy, as they can potentiate each other. Another factor can be that, as mentioned above, ETV shows the inhibition of AGR2 and a decrease in its connection with VEGF [30], while RPV inhibits VEGFR-2 [44]. These effects of each drug on different points of the VEGFs–VEGFRs pathway can be the cause of this high synergy between the two drugs, with them potentiating the effect of each other, with a greater effect and with less concentration the more time they are left to act on bladder cancer cells. The great effect of the combination at 72 h, while RPV alone at 72 h has a higher IC_50_ than at 48 h, shows further that its combination with ETV is truly advantageous for the treatment of bladder cancer.

ETV and DRV were the next best combination, but were only truly synergic at 48 h, as shown in both the cell viability graphs and in the synergy scores (Figure 15 and Table 2), beyond the concentrations of 10 µM each, which is interesting since both drugs had a lower IC_50_ at 72 h (Table 1). This drug combination is favorable for the treatment of HIV even in heavily penetrated patients [53]. The effect of ETV in apoptosis induction through the inhibition of CK1ε and the potential effect of DRV against LDHA that affects several of the other hallmarks of cancer can be working in tandem to make the combination of these drugs especially effective at 48 h time points. The last combination of DRV and RPV was almost not active on the UM-UC-5 cells, with almost no decrease in viability in comparison with each drug alone. Despite showing high synergy scores at 24 h, this is scarcely reflected in the cell viability graphs, with values never decreasing below 50% in viability, which makes sense when accounting for the lack of an IC50 for both drugs at that time point. At 72 h, in the concentrations between 0.1–1 µM, there was a significant decrease in cell viability in relation to each drug alone, and this coincides with the MSA, in which the Bliss–Loewe score indicates synergism, despite the overall combination being only additive, and 25 µM of each drug shows a 50% decrease in viability. This could be DRV also increasing the effect of RPV since its IC50 at 72 h is around 25 µM, which could be because these drugs have different mechanisms of action, since one is a PI and the other an NNRTI and could affect cancer cells at different time points.

This is a novel work as bladder cancer repurposing studies are very scarce and this disease still needs more efficient treatment methodologies, and while it is only a preliminary work, it has demonstrated great results. The use of DECREASE machine learning software with the SynergyFinder web application for the high-throughput screening presented is also a new way of studying a combination of drugs that will be further explored as well. In the future, it would be interesting to further study these drugs and combinations, particularly ETV and RPV and their combination, since these showed better promise in bladder cancer, mainly their mechanisms of action, to validate the theories presented in this study.

While our investigation into drug repurposing for bladder cancer treatment yielded insightful findings, it is essential to acknowledge the constraints inherent in our experimental design. The reliance on a single cell line may limit the generalizability of our results to diverse bladder cancer subtypes, emphasizing the need for future studies using a broader range of cell lines or patient-derived models to validate our findings across different contexts. Additionally, while our study proposes potential mechanisms of action for ETV, RPV, and DRV in bladder cancer cells, further experimental validation is required to confirm these hypotheses and elucidate the intricacies of their interactions within cellular pathways.

Advancements in technology, particularly Artificial Intelligence (AI), offer promising avenues for enhancing the diagnosis and treatment of bladder cancer. AI’s adaptability across various medical disciplines enables improved diagnostic accuracy, personalized treatment planning, and remote patient monitoring. By integrating patient data with clinical and multi-omic information, AI facilitates the identification of molecular signatures and biomarkers for predicting treatment responses. However, challenges such as regulatory approvals, the interpretability of machine learning models, and patient acceptance hinder its widespread clinical application. Addressing these limitations through prospective studies, regulatory clarity, and patient education is essential to fully harness the potential of AI in bladder cancer diagnosis and management [54].

## 4. Materials and Methods

### 4.1. Cell Culture

To evaluate the toxicity of rilpivirine (RPV), darunavir (DRV), and etravirine (ETV), human squamous cell bladder cancer cell lines UM-UC-5 were employed, since these cells present the most important features of the carcinoma of the bladder. The American Type Culture Collection (ATCC, Manassas, VA, USA) provided these cell lines, while Sigma-Aldrich (Merck KGaA, Darmstadt, Germany) provided the drugs. All reagents used were purchased from Millipore Sigma (Merck KGaA, Darmstadt, Germany), and cells were maintained in Dulbeco’s modified Eagle’s medium (DMEM) with a 10% fetal bovine serum (FBS), a 1% penicillin-streptomycin solution, in an incubation chamber at 37 °C and 5% CO_2_. Confluent cells were trypsinized for maintenance using 0.25% trypsin-EDTA (Gibco; Thermo Fisher Scientific, Inc., Waltham, MA, USA), which was followed by subculture in fresh DMEM media with 96 h intervals of medium renewal. For the experiments, 96-well plates were seeded with a density of 5000 UM-UC-5 cells per well (passages 150–153) that were left to adhere overnight. All equipment used in cell culture and treatments was previously sterilized, and work was performed in a sterilized laminar flow chamber with air filters, maintained and cleaned routinely, with all material doused with alcohol at 70% before entering the chamber.

### 4.2. Drug Treatment

The cytotoxicities of RPV, DRV, and ETV were evaluated alone using concentrations of 0.01, 0.1, 1, 10, 25, 50, and 100 μM after 24 h, 48 h, and 72 h. These concentrations are the standards used in our typical workflow when researching the repurposing of drugs, as they cover a wide range of concentrations and allow us to obtain IC_50_ values for our drugs. For the combination studies, the drugs were combined in pairs using the same concentrations for both drugs for 48 h. The negative control cells were treated with 0.1% of dimethyl sulfoxide (DMSO), which was the vehicle in which the drugs were dissolved. Each treatment was tested in three independent experiments.

### 4.3. Morphological Analysis

After the incubation time of the drugs, cell morphology was evaluated using a Leica DMI 6000B microscope with a Leica DFC350 FX camera (Leica Microsystems, Wetzlar, Germany). Images obtained were then analyzed using the Leica LAS X imaging software (v3.7.4) (Leica Microsystems, Wetzlar, Germany).

### 4.4. MTT Assay

Through the use of the MTT (thiazolyl blue tetrazolium bromide) colorimetric assay, the toxicity of the tested drugs and combinations was assessed. After the predetermined amount of time, 100 μL of a solution of 0.5 mg/mL of MTT in PBS (Sigma-Aldrich; Merck KGaA, Darmstadt, Germany) was added to each well. The MTT solution was removed from the cells after 2 h at 37 °C and 5% CO_2_ in complete darkness, and the purple formazan crystals that had formed were then solubilized in 100 μM of DMSO. Cell viability was determined by comparing the absorbance reads of the experimental groups with those of the negative control group using an automated microplate reader (Tecan Infinite M200, Tecan Group Ltd., Männedorf, Switzerland) that reads absorbance at 570 nm.

### 4.5. Statistical Analysis

The GraphPad Prism 9 system (GraphPad Software Inc., San Diego, CA, USA) was used to construct the cell viability graphs, and the data are displayed as the cell viability mean ± SEM. Dunnett’s multiple comparisons using one-way ANOVA were used to compare just the negative control and experimental drug groups. The viability findings of the combination experiments were compared with the viability results of each drug alone at the appropriate concentration using a two-way ANOVA. The threshold for statistical significance was *p* < 0.05.

The viability findings were first normalized to the viability of the negative control group and plotted with the logarithmized drug concentrations using a non-linear regression test to create the dose–response curves.

### 4.6. Drug Combination RESponse prEdiction (DECREASE)

To get the full matrix of combinations, the Drug Combination RESponse prEdiction (DECREASE) software was used (http://decrease.fimm.fi, accessed on 2 June 2023). This is a machine learning program that uses a limited amount of drug combination cell inhibition experiment data and predicts the whole combination matrix. This is done by implementing outlier measurements and the Non-negative Matrix Factorization algorithm (cNMF), and it is available for use under the GNU General Public License v3.0 [55]. For this, the percentage of inhibition values of all three drugs alone were input, as well as the pairwise combinations at the same concentrations (diagonal measurements).

### 4.7. Synergy Calculations

The full matrices obtained from the DECREASE method were then input into the SynergyFinder web application version 3.0 (https://synergyfinder.fimm.fi accessed on 2 June 2023). This applied synergy scoring models and automated outlier detection. The method chosen for this analysis combines the Bliss/Loewe consensus synergy, which combines the models for Bliss excess, Loewe additivity, and highest single agent (HAS), eliminating any false positive synergy results [56]. The results are shown as two-dimension synergy heat maps, where areas colored in red represent synergism, while green-colored areas show antagonism. The most synergistic area is also highlighted. The Bliss–Loewe synergy scores (δ-score) give the average excess response due to drug interaction and can be interpreted with less than −10 being a pairwise interaction that is likely antagonistic, between −10 to 10 being likely additive, and greater than 10 meaning the interaction between drugs is likely synergistic [57].

## 5. Conclusions

In conclusion, this study explored the potential of drug repurposing for the development of new cancer treatments, focusing on the antiretroviral drugs DRV, RPV, and ETV in bladder cancer cells. Among the drugs tested, ETV demonstrated the most promising results, exhibiting concentration and time-dependent effects on cell viability. The drug’s ability to inhibit casein kinase 1 ε (CK1ε) and the human anterior gradient protein 2 homolog (AGR2) may contribute to its efficacy in inducing cell cycle arrest and apoptosis in bladder cancer cells. RPV also showed potential, particularly at 48 h, possibly by inhibiting aurora A kinase and vascular endothelial growth factor receptor 2 (VEGFR-2), both of which play crucial roles in cancer progression and angiogenesis. On the other hand, DRV exhibited weaker effects, suggesting that longer exposure or further investigation of its mechanisms of action, such as its potential as a lactate dehydrogenase A (LDHA) inhibitor, may be necessary.

This study involved drug repurposing and drug combinations, revealing that the synergy between ETV and RPV was most pronounced, especially at 72 h, indicating enhanced individual effects. ETV and DRV exhibited synergistic effects at 48 h but were less effective at 72 h. Conversely, the combination of DRV and RPV demonstrated limited activity in the tested bladder cancer cells.

This research contributes to the sparse literature on drug repurposing in bladder cancer, emphasizing the potential of ETV and RPV, either individually or in combination, as viable treatment options. The utilization of machine learning software and high-throughput screening tools introduces an innovative approach to studying drug combinations.

Although this work serves as a preliminary investigation, it yields promising results, necessitating further exploration. Subsequent studies should concentrate on validating the proposed mechanisms of action, particularly for ETV and RPV, to gain a comprehensive understanding of their therapeutic potential in bladder cancer. Once this information is acquired, additional experiments should be undertaken using in vivo models to determine whether the effects observed in this study extend to living complex organisms, as this correlation is not always definitive. Elucidating the efficacy and safety profiles of these drug combinations in preclinical models paves the way for potential clinical trials aimed at evaluating their effectiveness in human subjects. The observed concentration and time-dependent effects underscore the importance of optimizing treatment regimens tailored to individual patient profiles, thus aligning with the principles of personalized medicine. Additionally, the identification of specific molecular targets opens avenues for targeted therapies and biomarker-driven approaches, enhancing treatment precision and patient outcomes.

## Figures and Tables

**Table 1:** Summarized values of IC_50_ for all drugs and time points.

Time	Darunavir (µM)	Rilpivirine (µM)	Etravirine (µM)
24 h	>100	>100	24.76
48 h	84.17	9.604	32.77
72 h	25.60	59.63	5.923

**Table 2:** Bliss–Loewe overall and MSA synergy scores for all the combinations at all time points. Values between −10 and 10 indicate additive results, while those higher than 10 indicate synergism.

Synergy	Time	DRV + RPV	ETV + RPV	ETV + DRV
Synergy score	24 h	25.186	16.886	7.247
	48 h	−4.285	15.694	17.712
	72 h	7.473	28.431	0.315
MSA synergy score	24 h	36.813	28.894	15.093
	48 h	3.277	25.986	28.835
	72 h	13.656	47.532	9.701

## Data Availability

Data are contained within the article.
